# Acute Effects of Tai Chi Training on Cognitive and Cardiovascular Responses in Late Middle-Aged Adults: A Pilot Study

**DOI:** 10.1155/2018/7575123

**Published:** 2018-01-31

**Authors:** Tiffany C. Y. Cheung, Karen P. Y. Liu, Janet Y. H. Wong, Young-Hyeon Bae, Stanley Sai-Chuen Hui, William W. N. Tsang, Yoyo T. Y. Cheng, Shirley S. M. Fong

**Affiliations:** ^1^School of Public Health, Li Ka Shing Faculty of Medicine, The University of Hong Kong, Pokfulam, Hong Kong; ^2^School of Science and Health (Occupational Therapy), Western Sydney University, Penrith, NSW, Australia; ^3^School of Nursing, Li Ka Shing Faculty of Medicine, The University of Hong Kong, Pokfulam, Hong Kong; ^4^Rehabilitation Clinical Research Center, Korea Worker's Compensation & Welfare Service Daegu Hospital, Daegu, Republic of Korea; ^5^Department of Sports Science and Physical Education, The Chinese University of Hong Kong, Shatin, New Territories, Hong Kong; ^6^Department of Rehabilitation Sciences, The Hong Kong Polytechnic University, Hung Hom, Hong Kong

## Abstract

This study explored the immediate effects of Tai Chi (TC) training on attention and meditation, perceived stress level, heart rate, oxygen saturation level in blood, and palmar skin temperature in late middle-aged adults. Twenty TC practitioners and 20 nonpractitioners volunteered to join the study. After baseline measurements were taken, the TC group performed TC for 10 minutes while their cognitive states and cardiovascular responses were concurrently monitored. The control group rested for the same duration in a standing position. Both groups were then reassessed. The participants' attention and meditation levels were measured using electroencephalography; stress levels were measured using Perceived Stress Scale; heart rate and blood oxygenation were measured using an oximeter; and palmar skin temperature was measured using an infrared thermometer. Attention level tended to increase during TC and dropped immediately thereafter (*p* < 0.001). Perceived stress level decreased from baseline to posttest in exclusively the TC group (*p* = 0.005). Heart rate increased during TC (*p* < 0.001) and decreased thereafter (*p* = 0.001). No significant group, time, or group-by-time interaction effects were found in the meditation level, palmar skin temperature, and blood oxygenation outcomes. While a 10-minute TC training could temporarily improve attention and decrease perceived stress levels, it could not improve meditation, palmar skin temperature, or blood oxygenation among late middle-aged adults.

## 1. Introduction

The world's population is aging at an increasing rate. In 2015, 900 million people were aged 60 years or older. By 2050, this number is expected to reach 2 billion, with 80% living in low- and middle-income countries [1]. As people age, their cognitive (e.g., attention and relaxation) and physical (e.g., cardiovascular) functions deteriorate, which can lead to more health-related problems. Electrophysiological and behavioral studies have reported that older adults are less able to sustain attention, which is associated with prefrontal cortex functional deficits [2]. They have furthermore showed that older adults are unable to switch attention between two motor tasks [3] and need to increase attentional demand in complicated motor tasks [4]. Stress could further compromise the cognitive functions of apparently healthy aging adults [5] as it increases the pituitary release of the adrenocorticotrophic-stimulating hormone, which controls the release of glucocorticoids (stress hormones). Long-term exposure to these stress hormones increases the death of neurons in the central nervous system and may contribute to hippocampal atrophy and eventually cognitive decline [6]. Thus, it is essential to explore effective interventions to reduce stress and improve the cognitive functions in aging (late middle-aged) adults.

In addition to cognitive decline, older adults also experience a decline in their health and physical functions. For example, it is well known that vascular elasticity (vasodilatory capacity) is reduced in the elderly and that this could lead to higher total peripheral vascular resistance, slower peripheral blood flow, and possibly hypertension [7]. Heart rate is also adversely affected as autonomic modulation decreases with age [8]. All these factors may negatively affect the cardiovascular functions and fitness of older adults. Effective exercise interventions are thus needed to slow down the deterioration of both the physical and cognitive functions of aging and late middle-aged adults [9].

Tai Chi (TC) is a low-impact, inexpensive, mind-body exercise that originated in ancient China. The training involves coordinated diaphragmatic breathing, with slow and graceful movements to achieve tranquility of the mind. The practice has become more popular around the world, especially among the elderly and medically compromised individuals [10]. Several studies have reported that TC training can improve cognitive functions (e.g., attention) [11, 12], promote relaxation and reduce stress [13, 14], and improve cardiovascular outcomes [15, 16]. However, many of these studies are cross-sectional in nature and thus cannot be used to establish cause-and-effect relationship [12, 15]. Furthermore, these studies did not measure the immediate effects of TC exercise [11, 13]. To the best of our knowledge, there has not yet been a study that has assessed the acute effects of TC training on the cognitive and cardiovascular outcomes of community-dwelling late middle-aged individuals.

This study aimed to explore the acute effects of TC exercise on attention and meditation, perceived stress level, heart rate, oxygen saturation level in blood, and palmar skin temperature in late middle-aged adults. It was hypothesized that TC can improve the cognitive states and cardiovascular responses of late middle-aged adults. Positive findings would suggest that TC could be an inexpensive intervention to improve cognitive and cardiovascular functions among the growing number of middle-aged and older people.

## 2. Materials and Methods

### 2.1. Study Design

This study was conducted as a prospective nonrandomized controlled intervention trial. The participants were allocated to either a TC group or control group according to whether they had any experience with TC or not.

### 2.2. Participants

The TC and control participants were recruited from two local TC associations and the community, respectively, by convenience sampling. The inclusion criteria for the participants were as follows: aged between 50 and 65, Chinese ethnicity, at least one year of TC experience (TC group), or no experience in TC or other martial arts (control group). The participants were excluded from the study if they had any major illnesses, such as uncontrolled hypertension, uncontrolled diabetes mellitus, cardiorespiratory diseases, significant musculoskeletal diseases, neurological disorders, or cognitive impairment. Written informed consent was obtained from all eligible participants before the trials commenced. The University of Hong Kong/Hospital Authority Hong Kong West Cluster's Institutional Review Board approved the study and all procedures were carried out in accordance with the Declaration of Helsinki.

### 2.3. Interventions

The participants in both groups were required to stand quietly for 1 minute before the intervention period (pretest) and another minute after the intervention (posttest). During the intervention period, the TC group participants performed TC individually for 10 minutes. They chose their preferred style of TC (Wu style that emphasizes small and compact movements or Yang style that emphasizes medium-sized and less compact movements) and exercised at their own pace. The exact TC postures/forms can be found in [17]. The control group participants were given no specific exercise instructions during the intervention period and were asked to stand quietly in a relaxed manner for 10 minutes.

### 2.4. Outcome Measurements

The interventions and assessments took place at two local community centers where the environmental temperature and relative humidity were monitored. The assessments for the participants in each group took 12 minutes and comprised a 1-minute standing baseline assessment (pretest), a 10-minute intervention period (TC or standing), and a 1-minute standing assessment (posttest). The participants' attention and meditation levels, heart rate, and oxygen saturation in the blood were measured continuously during the 12-minute assessment period. Perceived stress level and palmar skin temperature were measured at the pretest (first minute) and posttest (final minute). The measurement procedures were standardized and conducted by a trained student researcher who was not blinded to group assignment.

#### 2.4.1. Primary Outcome Measures

*(1) Attention and Meditation Levels*. During the intervention period, the participants' attention and meditation levels were measured by a single-channel electroencephalographic (EEG) device, Mindwave Mobile EEG headset (NeuroSky Inc., USA), and data acquisition software (NeuroView). The EEG device was validated in a previous study [18]. During the measurement, the participants wore the EEG headset with the active electrode placed on their left forehead (i.e., Fp1 position according to the International 10–20 System of electrode placement) and the reference electrode clipped to their left earlobe. Known noise frequencies, such as those caused by eyeblink, extraocular, or muscular activities, were automatically eliminated using proprietary algorithms. The sampling rate was 512 Hz. Raw brain wave signals were digitized and transmitted via Bluetooth to the NeuroView software installed on a laptop [18, 19].

The NeuroView software continuously recorded the EEG activity of the prefrontal cortex from pretest to posttest. It then converted the raw prefrontal cortex EEG signals to an attention index (0–100) and a meditation index (0–100) using Fast Fourier Transform and a preconfigured proportion of EEG alpha, beta, theta, and delta activities. The attention and meditation index were generated and recorded for every second of the EEG recording. Higher attention or meditation indices indicated a higher level of mental concentration or relaxation [18, 19]. The average attention and meditation indices for each time period (i.e., pretest, intervention, and posttest) were calculated and used for analysis.

*(2) Perceived Stress Level*. A 10-item Perceived Stress Scale (PSS) [20] was used to measure the participants' stress level. The participants were asked to fill in the questionnaire before and after the intervention period. The PSS scale is the most widely used psychological instrument for measuring the perception of stress in adults and demonstrates excellent psychometric properties [21]. It includes 10 questions that ask participants about their feelings and thoughts during the last month. The participants rated their levels of appraised stress on a 5-point Likert scale. A PSS score (0–40) was obtained by reversing the responses to four positively stated items and then summing across all the items. A higher PSS score indicated higher stress levels [20]. The participants' PSS scores before and after the intervention period were used for analysis.

#### 2.4.2. Secondary Outcome Measures

*(1) Heart Rate and Oxygen Saturation in Blood*. A fingertip pulse oximeter (Prince-100, Heal Force, Hong Kong) measured the cardiovascular outcomes from pretest to posttest. The device, with a measuring accuracy of <3% [22], was clipped to the right index finger of each participant. Oxygen saturation in blood (in %) and heart rate (in bpm) were measured 12 times throughout the assessment period, at 1-minute intervals. The participants' average oxygen saturation in blood and heart rate for each time period (i.e., pretest, intervention, and posttest) were calculated and used for analysis.

*(2) Palmar Skin Temperature*. Palmar skin temperature reflects the circulatory status of the hand (a distal body part) and was assessed while the participants were standing. The skin temperature was taken from the center of the palm (corresponded to acupuncture point PC8) of the dominant hand using an infrared thermometer (AR300 + Smart Sensor, Arco, Hong Kong). The thermometer had a −32°C–400°C measurement range with 2°C precision. Any moisture from the hand was removed before taking the measurement [23]. The participants' palmar skin temperatures before and after the intervention period were recorded and used for analysis.

#### 2.4.3. Demographic Data

Relevant demographic information about the participants' age, sex, height, and weight was recorded. Information about their TC experience was furthermore obtained from interviews. Body mass index (BMI) was calculated by the formula: body weight (kg)/body height (m)^2^. The International Physical Activity Questionnaire (short form) was used to obtain the physical activity level of each participant before the physical assessments.

#### 2.4.4. Statistical Analyses

Based on a previous study that showed that TC exercise can improve cognitive functions in older adults, with effect sizes ranging from 0.18 to 0.43 [24], this study expected a medium effect size of 0.25. Thus, a total sample of 34 participants was required to achieve 80% power with a two-tailed alpha value of 5%. Given an anticipated 20% dropout rate, the minimum required sample size was 41 participants (i.e., 20-21 participants per group).

Independent* t*-test (for continuous data) and chi-square test (for categorical data) were used to compare the demographic data and baseline outcome variables between the TC and control groups. If any significant differences between the two groups were identified at the pretest stage, the outstanding baseline outcome variables were entered as covariates in the subsequent analyses. Two-way repeated measures analysis of covariance (ANCOVA) was performed to test the effects of TC on the primary and secondary outcome measures. The between-subject factor was group and the within-subject factor was time. The intention-to-treat principle was implemented in any dropout cases. If the two-way ANCOVA test demonstrated a significant time, group, or group-by-time interaction effects for a particular outcome, post hoc analyses using paired* t*-tests (to detect within-group differences) or independent* t*-tests (to detect between-group differences) were carried out, as appropriate. The overall significance level was set at 0.05 (two-tailed) and was Bonferroni adjusted for the pairwise* t*-tests. Statistical analyses were performed using the Statistical Package for Social Sciences 20.0 software (IBM, Armonk, NY).

## 3. Results

Forty healthy middle-aged and older adults (20 TC practitioners and 20 nonpractitioners) volunteered to participate in the study. All the participants met the inclusion and exclusion criteria. Half of the TC practitioners (*n* = 10) practiced the Wu style TC and the other half of them (*n* = 10) practiced the Yang style TC. Their demographic characteristics are presented in Table 1. Because BMI and physical activity level differed between the TC and control groups at baseline, they were treated as covariates in the subsequent analyses. All the participants completed the study within a single visit. An intension-to-treat analysis was not needed as there were no dropouts or missing data. On the day of measurement, the average environmental temperature was 19.5°C and the relative humidity was 84.7%. No adverse events were reported during the assessments and intervention.

The group-by-time interaction effect was significant for the EEG-derived attention level (*p* = 0.006). In the TC group (*p* < 0.001), the analysis revealed that attention level tended to increase from the pretest to intervention period (*p* > 0.017) and drop from the intervention period to posttest. The control group demonstrated no within-group changes overtime (*p* > 0.05). In addition, there were no significant differences between the two groups at any time points (*p* > 0.05) (Table 2).

The EEG-derived meditation level results showed no group-by-time interaction effect (*p* = 0.203), group effect (*p* = 0.455), or time effect (*p* = 0.740). Group-by-time interaction effect (*p* < 0.001) and group effect (*p* < 0.001) were statistically significant for the perceived stress level. The post hoc analysis revealed that perceived stress level decreased by 25% in the TC group after the intervention (*p* = 0.005). Significant between-group differences were found at both the pretest and posttest (*p* < 0.05). The pretest between-group difference was adjusted for in the two-way ANCOVA test. At posttest, the perceived stress level was 44% lower in the TC group than in the control group (*p* < 0.001), indicating that stress could be reduced through TC training (Table 2).

The heart rate outcome increased in both the TC (*p* < 0.001) and control groups (*p* = 0.010) from pretest to intervention period. However, only the TC group showed a decrease in heart rate from intervention to posttest (*p* = 0.001). In addition, heart rate was higher in the TC group compared to the control group both during the intervention period (*p* < 0.001) and at posttest (*p* = 0.002). The exercise heart rate during TC in the TC group was 88.4 bpm, which was much higher than the control group (73.83 bpm, *p* < 0.001). Regarding other cardiovascular outcomes (oxygenation in blood and palmar skin temperature), the group-by-time interaction, group, and time effects were not statistically significant (*p* > 0.05) (Table 2).

## 4. Discussion

This study explored the acute effects of TC training on mental states in late middle-aged adults. The results showed that EEG-derived attention level tended to increase during TC training and dropped immediately after TC. This finding concurred with previous studies showing that TC could improve the attention of older adults [11, 12, 25, 26]. For example, Kim and colleagues [11] reported that mental-attentional vigilance improved after 16 weeks of TC training among Chinese participants. Kerr and colleagues [26] also suggested that TC practitioners had sharper tactile acuity compared to age-matched controls. This indicated that TC practitioners had higher specific attention on their extremities. Man and colleagues [12] reported a similar result and found that selective attention and mental flexibility were better among older TC practitioners compared to nonpractitioners. Hawkes and colleagues [25] attempted to provide an explanation for this phenomenon and suggested that because TC training requires moderate mental exertion, it could benefit the neural substrates of executive attention (response inhibition, updating of working memory, and mental set shifting). Indeed, as this study demonstrated, mental exertion (attention) increased during TC exercise. However, the improvement was temporary and reversible if the TC training duration was not long enough (10 minutes). Further study is needed to explore the optimal TC training duration required to sustain the beneficial effect on cognitive function, and in particular, attention.

The results also revealed that TC had no effect on EEG-derived meditation level in late middle-aged adults. The meditation level did not increase significantly even during TC practice. This result was unexpected as TC, a mind-body exercise, embodies meditation [27]. This finding may be because the TC practitioners in the study did not meditate or use TC sense, or simply due to the distraction induced by simultaneous EEG and heart rate recording during the practice [14]. In further studies, more experienced TC practitioners or TC masters may be recruited to ensure that they had meditated during training and probably increase the TC exercise duration to verify the results.

Although the meditation (relaxation) level did not change during and after the TC exercise, the perceived stress level decreased dramatically from baseline to posttest among the TC practitioners. This finding concurred with previous studies [13, 28, 29] and suggested that TC training can reduce physiological and psychological stress through certain physiological mechanisms, such as the general autoregulatory self-healing mechanism and endogenous autoregulatory signaling mechanism, which is linked to the brain's innate reward and motivation circuitries (limbic system) [13]. In addition, TC has intrinsic favorable effects on reducing stress as a kind of moderate-intensity exercise [29, 30]. TC practice requires practitioners to be focused and concentrated and ignore distractions and stressors. In this way, TC exercise could lead to inner peacefulness and less stress for practitioners [29].

Regarding the cardiovascular outcomes, heart rate increased while performing TC with an average exercise heart rate of 88.4 bpm and decreased immediately after TC. TC practitioners also had higher heart rate values compared to the control group during and after the exercise period. Nonetheless, the higher heart rate of TC practitioners does not necessarily mean that TC training for 10 minutes can improve participants' cardiovascular fitness as, according to the American College of Sports Medicine guidelines, this requires an optimal exercise heart rate of 64–94% of maximum heart rate [31]. Previous studies reported that the average heart rate values during TC training ranged from 115 to 140 bpm, corresponding to only 50.3–58.0% of heart rate reserve, among older adults [30, 32]. Lai and colleagues [15] also reported that the mean heart rate of their participants exceeded 70% of maximum heart rate during TC exercise. These authors thus concluded that TC is a moderate-intensity exercise that could improve cardiovascular fitness [15, 30, 32]. However, the average exercise heart rate of the TC participants in this study was only 88.4 bpm and the TC exercise intensity was too low compared to previous studies [15, 30, 32]. Thus, we postulated that a 10-minute TC exercise may be too short to achieve the usual TC exercise heart rate. During TC practice, the heart rate increased steadily in the first 8 to 10 minutes, and then it reached a nearly steady state until the end of the exercise [15, 30, 32]. Further study could incorporate longer TC duration (e.g., 30 minutes) and standardized TC style/form to control the overall TC training volume of the participants before measuring heart rate or other hemodynamic outcomes. Furthermore, our results revealed that the resting heart rate at baseline was higher in the TC group that that of the control group, though it was not significant statistically. Possible explanation is that the anticipation of exercise in the TC group may increase resting heart rate. Further study should include a longer resting period (e.g., 5 minutes) to minimize the difference of resting heart rate between groups at baseline.

The palmar skin temperature, which reflects the peripheral cutaneous microcirculatory function of the participants, did not increase after TC exercise. This was in contrast to previous studies that showed that TC training could enhance cutaneous microcirculatory function, skin temperature [33], and endothelium-dependent dilation in skin vasculature of older adults [34]. This discrepancy could be due to two factors. First, the TC exercise intensity (54.3% of maximum heart rate) in this study may have been too low to elicit any changes in peripheral blood flow and palmar skin temperature. Second, palmar skin temperature is closely related to onsets of focus, rest, and withdraw periods during TC practice and appears to be volitional activation of known vasomotor mechanisms [35]. The TC practitioners in this study did not meditate (relax) during the 10-minute exercise and so no vasomotor (palmar skin temperature) change was observed.

The findings showed that oxygen saturation in blood cannot improve through TC training. This concurred with a previous study suggesting that a 12-month Cheng style TC program cannot improve oxygenation in the blood of apparently healthy older individuals [36]. The major reason could be that the participants in both studies were healthy adults whose blood oxygenation levels were in the normal range before TC and thus further improvement may not be possible with TC training.

Although the results of this study were quite promising, some limitations should be noted. First, the participants were recruited by convenience sampling. Group assignment was not randomized and both assessor and participants were not blinded to the grouping. These issues may lead to self-selection bias and tester bias, which could confound the results. Further study should use a stratified randomized controlled study design (stratified by sex and other physical characteristics of the participants) and assess the participants 2 to 3 times with longer baseline measurement duration to reduce potential measurement errors. Second, the TC styles performed by the participants were not standardized. Different styles and forms of TC may affect cognitive and cardiovascular outcomes differentially [10, 16]. Further study should standardize the TC intervention and use a longer measurement period (e.g., 30 minutes). Third, the temperature and humidity varied from day to day in an uncontrolled community setting. This may confound the test results especially the palmar skin temperature. Future study may collect data in a well-controlled laboratory environment instead. Fourth, attention level improved and perceived stress level decreased for only around 10 minutes in the TC group; such temporarily improvements may not be clinically meaningful or important for cognitive function. Fifth, as all the participants in this study were healthy late middle-aged adults, the results cannot be generalized to diseased populations. Finally, this study only examined the immediate effects of TC exercise and the longer-term effects of TC on cognitive states and cardiovascular fitness remain unknown. Future randomized controlled trials are needed to confirm the long-term beneficial effects of TC and extract the therapeutic elements of TC before fully implementing it in the stress reduction programs or cardiovascular fitness training programs of community-dwelling middle-aged and older adults.

## 5. Conclusions

TC training temporarily improved attention and decreased perceived stress levels but did not improve meditation, palmar skin temperature, or oxygen saturation level in blood. Thus, while TC could be a potential mind-body exercise to improve attention and reduce stress among late middle-aged adults, repeated practice may be needed to sustain the beneficial effects. Further study is needed to confirm the long-term effects of TC on cognitive and cardiovascular functions of community-dwelling late middle-aged adults.

## Figures and Tables

**Table 1:** Participants' characteristics.

	Tai Chi group (*n* = 20)	Control group (*n* = 20)	*p* values
Age (years)	57.2 ± 4.9	56.6 ± 5.4	0.691
Sex (*n*)	13 males and 7 females	6 males and 14 females	0.068
Weight (kg)	63.5 ± 11.7	55.8 ± 10.1	0.033^*∗*^
Height (m)	1.6 ± 0.1	1.6 ± 0.1	0.789
Body mass index (kg/m^2^)	23.4 ± 2.9	20.7 ± 2.1	0.002^*∗*^
Physical activity level (MET minutes per week)	3336.7 ± 3227.9	1636.4 ± 1360.3	0.039^*∗*^
Tai Chi experience (years)	6.4 ± 3.6	—	—

Means ± standard deviations are presented unless specified otherwise; *∗* denotes a difference significant at *p* < 0.05.

**Table 2:** Outcome measurements between the two groups (pretest, during intervention and posttest) and within individual groups.

	Tai Chi group (*n* = 20)	Control group (*n* = 20)	Group × time interaction effect	Group effect	Time effect
	Mean ± SD	Mean ± SD	*p* value	*p* value	*p* value
*Cognitive states*					
Attention level					
Pretest	54.27 ± 14.03	48.55 ± 16.45	0.006^*∗*^	0.112	0.323
Intervention	60.01 ± 9.24	45.86 ± 9.43			
Posttest	48.35 ± 10.26^b^	48.04 ± 14.37			
Meditation level					
Pretest	60.24 ± 14.70	58.06 ± 14.53	0.203	0.455	0.74
Intervention	67.03 ± 9.88	58.38 ± 7.24			
Posttest	57.41 ± 12.85	56.22 ± 7.50			
Perceived stress level					
Pretest	11.40 ± 7.24^d^	15.50 ± 4.30	<0.001^*∗*^	<0.001^*∗*^	0.626
Posttest	8.55 ± 5.87^c,d^	15.35 ± 3.47			
*Cardiovascular responses*					
Heart rate (beats per minute)					
Pretest	78.15 ± 11.82	71.25 ± 10.27	<0.001^*∗*^	0.003^*∗*^	0.002^*∗*^
Intervention	88.40 ± 11.99^a,d^	73.83 ± 7.63^a^			
Posttest	82.95 ± 10.67^b,d^	73.25 ± 7.65			
Oxygen saturation in blood (%)					
Pretest	97.65 ± 1.39	97.15 ± 1.73	0.382	0.290	0.523
Intervention	97.85 ± 0.92	97.91 ± 1.13			
Posttest	97.95 ± 1.32	98.05 ± 1.23			
Palmar skin temperature (°C)					
Pretest	29.42 ± 3.94	29.92 ± 2.49	0.192	0.098	0.046
Posttest	29.69 ± 3.91	29.98 ± 2.77			

*Group by Time Interaction, Group and Time Effects*. *∗* denotes a difference significant at *p* < 0.05. *Within Group*. a denotes a difference significant at *p* < 0.017 when compared with pretest values; b denotes a difference significant at *p* < 0.017 when compared with intervention values; c denotes a difference significant at *p* < 0.05 when compared with pretest values. *Between Groups*. d denotes a difference significant at *p* < 0.05 when compared with the control group.

## Abbreviations

TC: Tai Chi
EEG: Electroencephalography
PSS: Perceived Stress Scale
BMI: Body mass index
ANCOVA: Analysis of covariance.
